# A Deep Learning Approach for Precision Viticulture, Assessing Grape Maturity via YOLOv7

**DOI:** 10.3390/s23198126

**Published:** 2023-09-27

**Authors:** Eftichia Badeka, Eleftherios Karapatzak, Aikaterini Karampatea, Elisavet Bouloumpasi, Ioannis Kalathas, Chris Lytridis, Emmanouil Tziolas, Viktoria Nikoleta Tsakalidou, Vassilis G. Kaburlasos

**Affiliations:** 1 Human-Machines Interaction Laboratory (HUMAIN-Lab), Department of Computer Science, International Hellenic University (IHU), 65404 Kavala, Greece; evbadek@cs.ihu.gr (E.B.); kalathas@emt.ihu.gr (I.K.); lytridic@cs.ihu.gr (C.L.); tzioman@abo.ihu.gr (E.T.); vitaaka@cs.ihu.gr (V.N.T.); 2 Department of Agricultural Biotechnology and Oenology, International Hellenic University, 66100 Drama, Greece; ekarapatzak@gmail.com (E.K.); katerina_karampatea@yahoo.gr (A.K.); elisboul@abo.ihu.gr (E.B.)

**Keywords:** grape maturity detection, object detection, maturity estimation, YOLO

## Abstract

In the viticulture sector, robots are being employed more frequently to increase productivity and accuracy in operations such as vineyard mapping, pruning, and harvesting, especially in locations where human labor is in short supply or expensive. This paper presents the development of an algorithm for grape maturity estimation in the framework of vineyard management. An object detection algorithm is proposed based on You Only Look Once (YOLO) v7 and its extensions in order to detect grape maturity in a white variety of grape (Assyrtiko grape variety). The proposed algorithm was trained using images received over a period of six weeks from grapevines in Drama, Greece. Tests on high-quality images have demonstrated that the detection of five grape maturity stages is possible. Furthermore, the proposed approach has been compared against alternative object detection algorithms. The results showed that YOLO v7 outperforms other architectures both in precision and accuracy. This work paves the way for the development of an autonomous robot for grapevine management.

## 1. Introduction

The wine industry is facing several challenges, including labor shortages, rising labor costs, and the need for consistent, high-quality wine production. To address these challenges, the use of autonomous robots [1,2,3,4,5] in grapevines has emerged as a promising solution. By automating tasks such as pruning and harvesting, autonomous robots can potentially perform these tasks more precisely and consistently than human labor, resulting in better-quality grapes and more consistent wine production, enhancing the reputation and competitiveness of wineries. By integrating sensors and cameras, autonomous robots can collect and analyze data on grape health, yield, and maturity [6,7], providing valuable insights for grape growers and enabling them to make more informed decisions about vineyard management. 

Fruit maturity encompasses a complex interplay of biochemical, physiological, and physical changes that occur throughout the growth and development of the fruit. These changes include alterations in color, texture, flavor, aroma, and nutritional content. Historically, the assessment of fruit maturity relied on traditional methods such as visual inspection, touch, and taste. Advances in technology have revolutionized fruit maturity estimation, enabling more precise and efficient measurements [8,9,10,11,12]. Machine learning and artificial intelligence algorithms have further elevated the accuracy and reliability of fruit maturity estimation.

Cardellicchio et al. [13] focus on the utilization of artificial intelligence algorithms, particularly single-stage detectors rooted in YOLOv5, for the identification of phenotypic characteristics in tomato plants. The objective is to recognize nodes, fruit, and flowers within demanding datasets stemming from a stress experiment featuring various tomato genotypes. The outcomes indicate that these models achieve commendable accuracy, even in the face of obstacles such as varying object sizes, object resemblance, and color disparities in the input images. An enhanced YOLOv5n model, named I-YOLOv5n, is designed for the recognition and localization of mature cherry tomatoes [14]. The model incorporates a CA module to reduce background interference, utilizes the WIoU loss function, and employs a dynamic nonmonotonic focusing mechanism for accurate bounding box regression. It is memory-efficient at 4.4 MB, and suitable for real-time applications like automated cherry tomato picking. Detecting apple fruitlets is challenging due to factors like complex growth conditions, variable lighting, clustering, and overlapping fruitlets, particularly when they closely resemble the background. To address this, the study [15] utilizes a channel-pruned YOLO V5s deep learning algorithm with a compact model size. The process involves creating a detection model using transfer learning, followed by applying a channel pruning algorithm to simplify the model while maintaining detection efficiency. Experimental results demonstrate the effectiveness of the channel-pruned YOLO V5s model in detecting apple fruitlets under diverse conditions. It achieves impressive metrics, including a recall of 87.6%, precision of 95.8%, F1 score of 91.5%, and a low false detection rate of 4.2%. Additionally, the model operates swiftly at an average detection time of 8 milliseconds per image and maintains a small model size of only 1.4 megabytes. Focusing on YOLO architecture, ALAD-YOLO, is a lightweight network for accurate and fast detection of apple leaf diseases [16]. It builds on YOLO-V5s with a Mobilenet-V3 backbone, reducing computational cost. Innovative modules like DWC3-ghost, SPPCSPC_GC, and CA attention enhance feature fusion, adaptability to resolutions, and target focus. ALAD-YOLO achieves 90.2% accuracy with 6.1 GFLOPs, surpassing existing models in both accuracy and efficiency, making it suitable for real-time apple leaf disease detection. Comparable lightweight architectures have been implemented in the YOLO framework by Wang et al. [17] and Liu et al. [18].

Sozzi et al. [19] evaluate six versions of the YOLO object detection algorithm for real-time grape bunch detection. White grape varieties are specifically targeted due to the complexity of identifying white berries against leafy backgrounds. YOLOv5x and YOLOv4 achieved impressive F1 scores of 0.76 and 0.77, operating at speeds of 31 and 32 FPS. YOLO5s and YOLOv4-TINY reached F1 scores of 0.76 and 0.69, respectively, at significantly faster speeds of 61 and 196 FPS. YOLOv5x, considering bunch occlusion, accurately estimated bunches per plant with a 13.3% average error. YOLOv4-TINY offered the best balance between accuracy and speed, making it a strong candidate for real-time grape yield estimation. YOLOv3 displayed a trade-off between false positives and false negatives, impacting the root mean square error (RMSE).

Early research on grape maturity primarily centered around chemical analysis methods [20]. Rabot et al. [21] focus on characterizing the phenolic maturity of grape seeds for three varieties by combining macroscopic analysis and biochemical analysis of tannins at relevant phenological stages. 

Grape maturity detection is an important task in the wine industry as it helps to determine the optimal time for harvesting grapes for wine production. Object detection [22] using computer vision has emerged as a promising technique for detecting grape maturity as it allows for the identification and classification of individual grape berries in images. Another method of object detection through convolutional neural networks (CNNs) is to identify grape berries within images, followed by image segmentation to isolate them from the background [23].

Qiu et al. [24] propose an algorithm utilizing improved YOLOv4 for grape maturity detection for red grape varieties and visual pre-positioning to guide grape-picking robots, achieving a remarkable accuracy of 93.52% and swift detection time. Predicting grape maturity in greenhouse conditions involves employing various indicators and the BPNN method, yielding recognition accuracies of up to 87% and demonstrating the effectiveness of multi-factor prediction over single-factor approaches [25]. 

To assess sugar content in Red Globe grapes, Jia et al. [26] introduce the FNRR framework, employing computer vision and deep learning to address imbalanced sugar content distribution and achieve high accuracy in the detection of the final stage of maturity. 

Using estimated in-field maturity indices [27] to guide grape harvesting can reduce costs associated with pre-harvest sampling and chemical analysis, as well as post-harvest storage and waste. To estimate the maturity indices of grape clusters, color imaging and the Intervals’ Numbers (INs) technique are combined. A neural network regressor is used to predict total soluble solids, titratable acidity, and pH based on the IN representation of CIELAB color space. The performance of the model is evaluated on *Vitis vinifera* cv. Assyrtiko cultivar images and the results show that the IN’s NN regressor is a promising tool for non-destructive and efficient assessment of grape maturity that can be integrated into an autonomous harvesting robot [28]. These studies collectively showcase the transformative potential of advanced technologies in accurately estimating fruit maturity, leading to improved harvesting practices and enhanced product quality.

The research review above addresses the state of the art in maturity estimation for various crops. Few research papers have been published on object detection and maturity estimation in the context of grape analysis. One such study by [24] concentrates solely on red grapes using YOLOv4, while another paper by [10] primarily detects grape bunch maturity without distinguishing between different maturity levels. The objective of this paper is to provide an accurate method of determining the maturity of grapes, as well as to ensure that the method is appropriate for use with an agricultural robot in the field and introduces a novel dimension by focusing on the maturity levels of the Assyrtiko cultivar, a white grape variety. This paper presents an accurate approach to grape maturity assessment. Utilizing the YOLO and Detectron 2 models, this study optimizes parameters and demonstrates the effectiveness of dataset augmentation. Training phases involving two, three, and five classes reveal the superiority of YOLOv7-X and YOLOv7-TINY in object classification and localization. The results indicate there is room for further enhancement and emphasize the models’ potential for improving object detection for agricultural applications.

The paper is structured as follows: Section 2 presents the dataset used in the experiments, and the methodology is described. The experimental results are presented in Section 3, and in Section 4 the results are discussed. Finally, in Section 5, conclusions are drawn and proposals for future work are made.

## 2. Materials and Methods

### 2.1. Dataset

**Figure 1:**
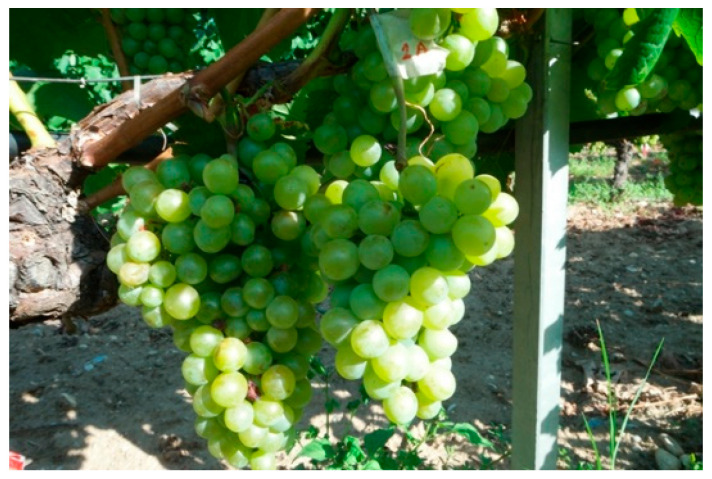
Grape samples with different illuminations.

To evaluate the effectiveness of a grape maturity detection system using computer vision, a diverse and comprehensive dataset of grape images was compiled. The dataset was carefully curated to include the *Vitis vinifera* cv. Assyrtiko grape variety, which is widely cultivated in Greece and, more recently, in other wine-producing regions like Australia. The images were obtained from grapevines during the ripening period in Greece when the grapes were at different stages of maturity. Specifically, the images were captured in a privately owned vineyard of 60 ha (Ktima Pavlidis, 41.200400 N, 23.953084 E, altitude 200 m) located in the region of Drama, which produces white, rosé, and red wines that best express the characteristics of the terroir and are recognized for their high quality. The vineyards of the study are in an area of terraced hills under Mediterranean climatic conditions with continental features. The topsoil type is of medium type, ranging from sandy loam to loamy clay. The camera used to obtain these pictures was a Samsung NX500, which has a high resolution of 6480 × 4320 pixels. The camera was positioned at a fixed distance from the grapevines to ensure consistency in image quality and size (Figure 1).

**Figure 2:**
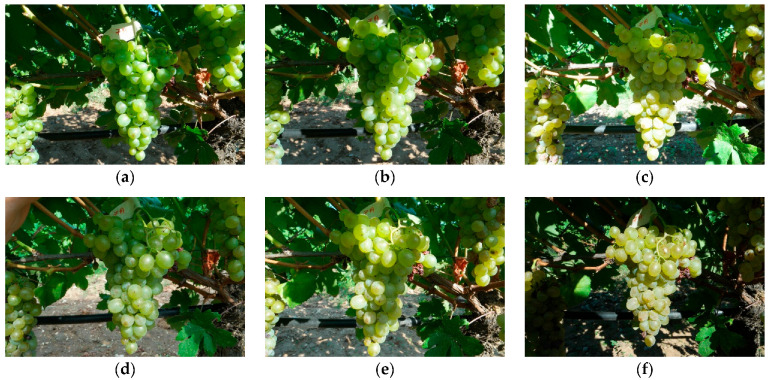
Maturity stage of white grape bunch 7 variety Assyrtiko (**a**) maturity level 1 (A1), (**b**) maturity level 2 (A2), (**c**) maturity level 3 (A3), (**d**) maturity level 4 (A4), (**e**) maturity level 5 (A5), and (**f**) maturity level 6.

To generate the dataset, ten grape clusters were selected. Ten photos of each grape cluster from different angles and lighting conditions were taken to ensure a diverse range of data. Figure 2 shows six photos of the same grape cluster captured on different dates (each week). A1 corresponds to week 1, A2 to week 2, and so on until A5, which represents week 5, the final stage of grape ripeness. This system helps track the progression of grape ripening over time, with A5 being the point at which the grapes are fully ripe and ready for harvest. Having a classification system for grape ripeness stages provides essential information for harvest optimization and rational decision making. Based on the above, it is ensured that grapes are harvested at the right time, leading to higher-quality products and improved agricultural management practices.

**Figure 3:**
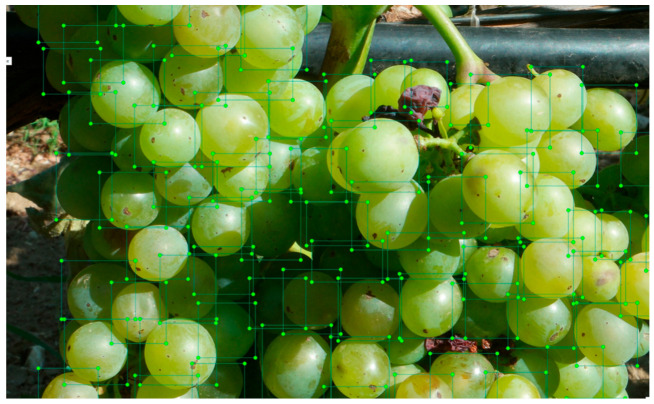
Labeling healthy berries of Assyrtiko variety.

In the vineyard area, the labeling process considers the scattered distribution of grapes, which may not necessarily form a bunch. It is important to consider cases where there are injuries or deformities on the ridges of the bunch, as they can impact the appearance of the entire image. Additionally, it might be challenging to accurately identify and evaluate all the berries in a low-illumination photograph. However, even with a few samples, valuable findings can still be obtained. Furthermore, if all the grapes can be located within an image, it becomes possible to estimate their quantity. Moreover, if the bounding boxes of the grapes are positioned closely together, it can be inferred that they form a bunch. These factors and considerations contribute to the accurate labeling and analysis of grape images in the vineyard. To ensure the accuracy and quality of the dataset, each image was carefully annotated with the corresponding grape maturity level, using a consistent and standardized approach. By labeling each individual grape berry, (Figure 3) the algorithm can identify any grapes in the bunch that may not be easily noticeable by human inspection. This can help in determining the overall quality of the grape bunch and in identifying the weight of the bunch based on the number of healthy grapes. Additionally, for ground truth annotation, labeling each grape berry allows for more accurate and precise predictions as compared to labeling only the grape bunch. It enables the algorithm to identify the specific location of the grape and provide more detailed information about the grape bunch. This information can be useful in improving the quality of the grape bunch and optimizing the harvesting process. *LabelImg* v1.8.1 is a widely used software for image annotation, an essential step in training computer vision models [25].

**Figure 4:**
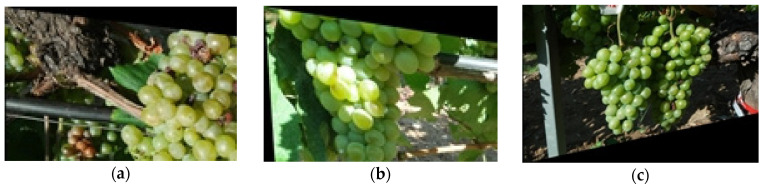
Data augmentation with (**a**) rotation, (**b**) crop, and (**c**) shear.

After collecting the images, the data were prepared for the model by augmenting the images (Figure 4) through cropping and shearing, which allowed the generation of close-resolution data for use with YOLO. This is because most YOLO (except YOLOv5 and YOLOv8 [29]) models require only 640 × 640 resolution, and augmenting the images helped generate enough data for the model to be trained effectively. The impact of resolution limits on training data in YOLO is multifaceted. It affects object detection precision, label density, object size distribution, and the need for data augmentation. Overall, the careful selection of grapevine cultivars, the high-resolution camera, and the diverse range of images captured, coupled with the data augmentation techniques, helped generate a comprehensive and diverse dataset for the proposed grape maturity detection model.

The images were then labeled according to their respective maturity levels. The dataset was split into training, validation, and testing sets, with 70%, 15%, and 15% of the images, respectively.

### 2.2. Yolo v7

You Only Look Once (YOLO) [30] is a state-of-the-art object detection algorithm that has been widely adopted in the computer vision community. The YOLO algorithm works by dividing the input image into a grid and predicting the object classes and bounding box coordinates for each grid cell. This approach enables YOLO to detect multiple objects in a single pass, making it more efficient than other object detection algorithms.

YOLOv7 [3] is an advanced version of the popular YOLO object detection algorithm. Its success has led to the development of several variants, including YOLOv2 [31], YOLOv3 [32], YOLOV4 [33] and YOLOv5, each with improved performance and features. Like its predecessors, YOLOv7 is a real-time object detection system that can detect and localize objects in images and videos with high accuracy and speed. However, YOLOv7 comes with several advantages over the previous versions of YOLO, which makes it a better choice for many real-world applications.

One of the most significant advantages of YOLOv7 over the previous versions is its improved detection accuracy. YOLOv7 improves upon previous versions by enhancing layer efficiency, incorporating effective model scaling techniques, utilizing re-parameterization strategies, and introducing an auxiliary head with a coarse-to-fine supervision approach. These advancements collectively contribute to better performance, efficiency, and adaptability in object detection tasks compared to earlier YOLO versions.

### 2.3. Performance Metrics

To evaluate the training performance in the experiments presented in this paper, a collection of performance metrics has been applied.

*IOU* stands for “Intersection over Union”, which is a measure of the overlap between two bounding boxes in object detection tasks. It is used to evaluate the accuracy of object detection models by calculating the ratio of the intersection area between the predicted bounding box and the ground truth bounding box to the union area of both boxes. A higher *IOU* score indicates better accuracy in object detection (Equation (1)).

(1) $$IOU=area\left(B_{p}\cap B_{g}t\right)/area\left(B_{p}\cup B_{g}t\right)$$

TP (true positives), FP (false positives), and FN (false negatives) are key terms used in evaluating object detection models. TP represents the correctly detected objects by the model, meaning the objects that were both predicted and labeled correctly. FP refers to the objects that were incorrectly predicted by the model as positive, i.e., false alarms or incorrect detections. FN denotes the objects that were missed by the model, meaning they were present in the ground truth but not detected by the model. These metrics provide crucial information about the model’s performance: TP reflects its accuracy in identifying objects correctly, FP indicates the rate of incorrect or false positive detections, and FN highlights the model’s ability to avoid missing objects or FP. A precision–recall (PR) curve is a graphical representation that illustrates the relationship between precision and recall for a given object detection model. It plots precision on the *y*-axis and recall on the *x*-axis. Average precision (AP) is a widely used metric in object detection that summarizes the PR trade-off of a model. To compute AP, a PR curve is generated by adjusting the confidence threshold for object detection. AP is often accompanied by mean average precision (mAP), which averages AP values across multiple classes or scenarios.

COCO (common objects in context) metrics [34] are widely used for evaluating object detection and instance segmentation models. Pascal metrics are commonly [35] used for evaluating object detection models on the Pascal VOC (visual object classes) dataset. The primary Pascal metric is the AP.

### 2.4. Methodology

The annotated dataset was used to train the YOLO and Detectron 2 models, where the models’ parameters were optimized to accurately predict the bounding boxes and class labels of berries in unseen images. After training the models, a model selection process was applied. By following this methodology, starting with dataset annotation, training the YOLO model, selecting the best-performing model, and measuring object detection metrics, it is possible to develop and evaluate an effective object detection system for detecting the maturity of berries in images.

## 3. Results

### 3.1. Training

For the experiments, a desktop computer with an Intel Core (TM) i7-9700 CPU was utilized, running at 3.00 GHz, 32.0 GB of RAM, and an NVIDIA GeForce RTX 3060 graphics card with 12 GB of memory. The experiments were conducted using Anaconda as the environment manager and PyTorch to implement the YOLOv7 architecture.

Apart from YOLO and its extensions, Detectron 2 was used to evaluate the performance of five classes. Detectron 2 is an advanced computer vision research platform developed by Facebook AI Research (FAIR), including Faster R-CNN, Mask R-CNN, and RetinaNet, among others. To train a YOLO model for grape maturity detection, each grape maturity level was assigned a numeric value from 1 to 5, based on the corresponding measurement data.

The YOLO model was first tested on a subset of the dataset consisting of white cv. Assyrtiko grapes. Ten grape bunches were selected from the 10 August visit, and each berry in these bunches was labeled with the appropriate maturity level class. More specifically, each berry was labeled as class A1 if it corresponded to maturity level 1 and class A5 if it corresponded to maturity level 5 (6 September). The model was then tested on a set of images of white cv. Assyrtiko grapes. 

Initially, the training process involved two classes. Subsequently, an additional class, A3, was introduced, and the model was retrained to accommodate this middle class. Finally, the full dataset, including all five classes, was used for training the model.

**Figure 5:**
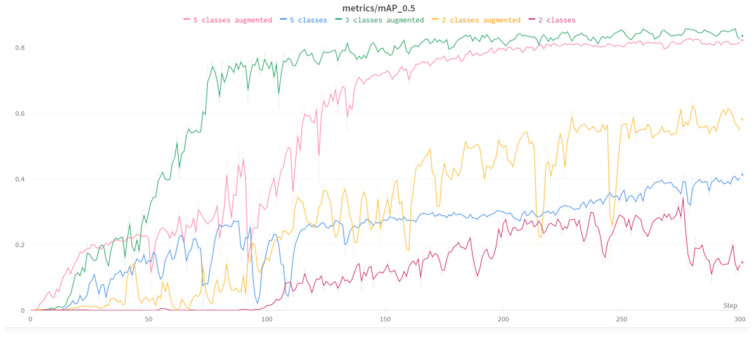
mAP_0.5 of a subset of 2–3, and 5 classes.

Ιn Figure 5 below, the metric mAP of 2 classes, 3 and 5 classes is depicted. Also, on some occasions, the dataset is augmented. The augmented dataset shows a significant flow in the training process compared with the simple dataset in the measurement of the mAP of training. After experimenting with combinations of batch size and epochs, 300 epochs in training with 12 batch sizes in each YOLOv7 model were determined to be appropriate.

**Figure 6:**
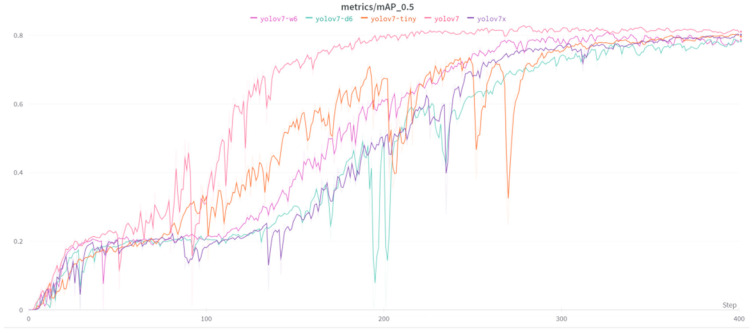
mAP_0.5: YOLOv7, X, D6, W6, and TINY.

In Figure 6, the value ranges from 0 to 1, with higher values indicating better performance. In this case, the mAP_0.5 ranges from around 0.2 to 0.8 during training, indicating that the model’s YOLOv7 and its extension accuracy are improving over time. 

**Figure 7:**
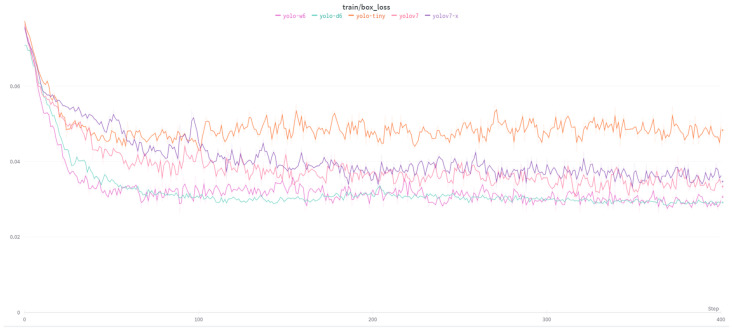
Train/box loss of YOLOv7, and its extensions.

Figure 7 shows the loss of the model during training for the localization (bounding box) task of the full dataset with augmentation. The performance value ranges from 0 to 1, with lower values indicating better performance. In this case, the box loss decreases over time, indicating that the model is improving at localizing objects in the images. The results indicate that the YOLO model results in increased accuracy over the course of training, as indicated by the decreasing losses and increasing mAP scores. However, the performance is not yet optimal, as indicated by the relatively low mAP scores and PR values. Further tuning of the model and training parameters may be necessary to improve performance.

### 3.2. Detection Measures

#### 3.2.1. Subset of Two Classes

**Figure 8:**
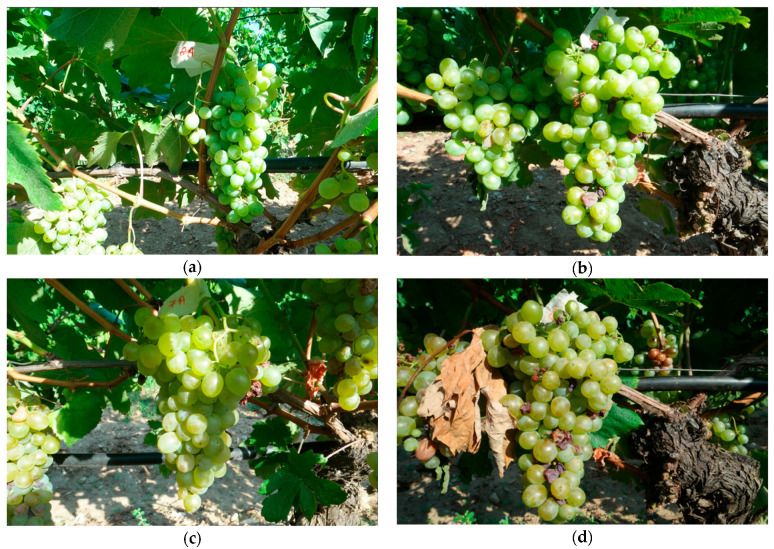
Test Images (**a**) Img 1 A1, (**b**) Img 2 A1, (**c**) Img 3 A5, and (**d**) Img 4 A5.

For the four pictures in Figure 8, their respective detection results have been analyzed using the YOLO algorithm.

As seen in Table 1 below, training sessions were executed using two distinct subsets: Subset A, comprising original images, and Subset B, wherein training was conducted utilizing the YOLOv7 model alongside augmented data, as previously detailed in Section 2.1. Table 1 presents performance metrics for two versions, A and B, using COCO evaluation criteria. Version B outperforms version A across all metrics, demonstrating improved object detection and localization accuracy. The higher values in AP, AP50, AP75, APmedium, APlarge, and AR100 for version B indicate its superior performance compared to version A. It appears that the YOLOv7 model was able to detect objects in the test images with a confidence threshold of 0.5. The results show that some images contain multiple A5 objects, while others have only one or none.

Table 2 illustrates Pascal metrics for versions A and B. Version B exhibits significantly higher performance across all metrics, showcasing substantial improvement in object classification and localization. The considerable increase in A1, A5, and mAP values for version B underscores its superior accuracy compared to version A in Table 2.

**Figure 9:**
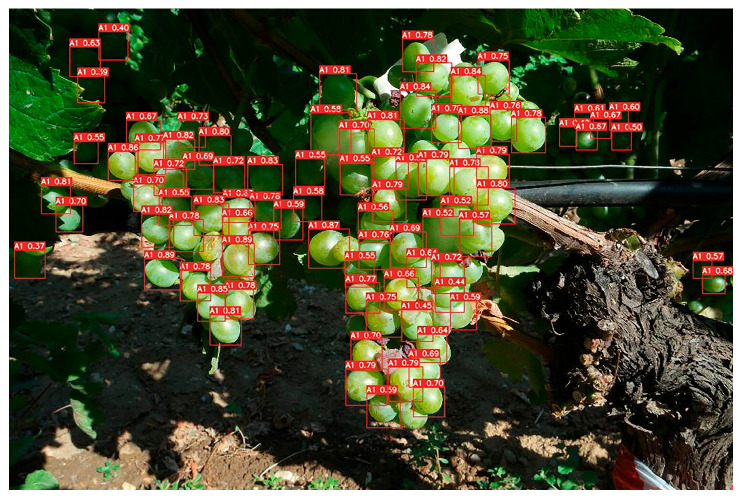
Detection of A1 grapes.

Figure 9 depicts a detection example from the training of version B. Bounding boxes, generated using the YOLOv7 model at a confidence threshold of 0.5, are overlaid on the image to showcase detected objects.

**Figure 10:**
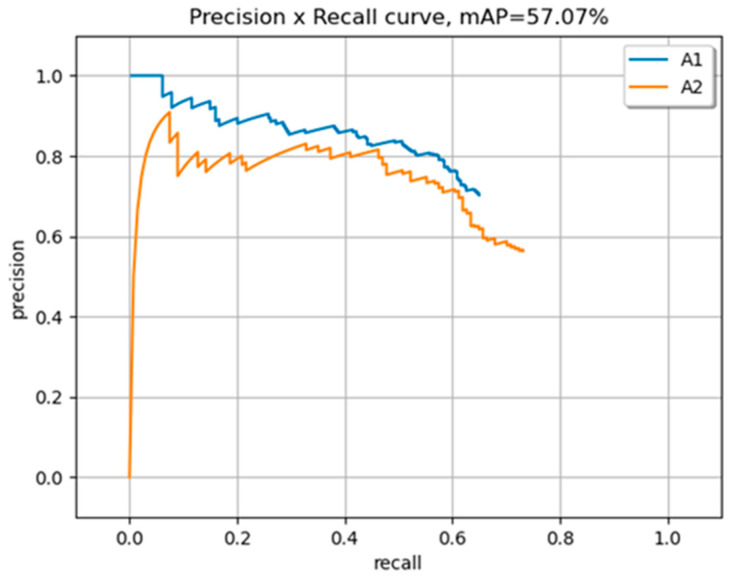
The mAP of two classes.

It is important to note that the model’s performance varies based on the complexity of the test images and the number of objects in each class. In terms of giving attention to most classes, it depends on the specific use case and what classes are most important to detect accurately. The mAP for both classes is 0.57. In Figure 10, the PR curve of version B’s training gives the result of mAP. No modifications were made to the initial subset of 62 training images, except for initially testing a set of four images from the test set.

Based on these metrics, it can be concluded that there is room for improvement in the detection and localization performance, especially for small objects. Further refinement and optimization are necessary to enhance the overall performance.

#### 3.2.2. Subset of Three Classes

Various packet modification techniques were tested in this case to achieve the presented results, with the best metric obtained being as follows. Subset A used for training consisted of 100 images, while 25 images were allocated for validation and another 25 for testing. It is important to note that the 25 test images did not contain the training bunches, focused on classes A1, A3, and A5. Subset B consists of 300 augmented images.

Table 3 presents COCO metrics for two versions. The second version outperforms the first across most metrics, indicating enhanced object detection and localization. The notably higher values in AP, AP50, AP75, APmedium, APlarge, AR10, and AR100 for the second version suggest its superior performance compared to the first.

Table 4 below shows the Pascal metrics for versions A and B. Version B demonstrates notable improvement across all metrics compared to version A, indicating enhanced object classification accuracy and localization. The significant increase in A1, A3, A5, and mAP values for version B underscores its superior performance in comparison to version A. In terms of the Pascal metric, classes A1, A3, and A5 achieve high AP values. The mAP for the Pascal metric is noted as 0.792. 

**Figure 11:**
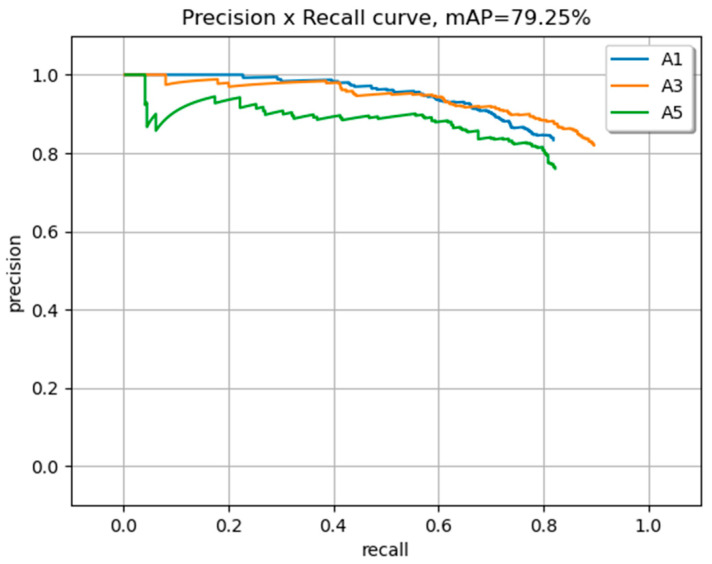
The mAP of three classes.

In Figure 11, the chosen YOLOv7 model trained on an augmented dataset is highlighted. The PR curve illustrates the performance characteristics of three classes.

**Figure 12:**
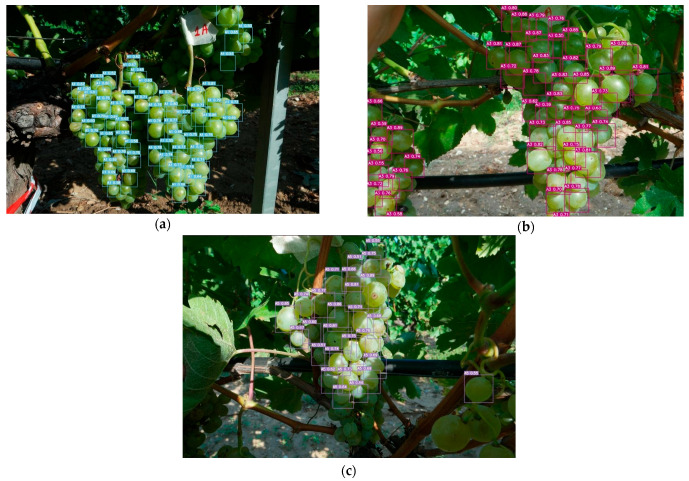
(**a**) Class A1, (**b**) Class A2, and (**c**) Class A5.

Based on the mentioned metrics, it can be concluded that the packet modification techniques applied in this study have resulted in improved detection and localization performance. The achieved AP and mAP scores indicate successful object detection across different classes. Detection images of each class are shown in Figure 12. Grape bunches that were in the test dataset are not included in the training and validation dataset.

Further analysis and refinement of the techniques can lead to even better results. Overall, these findings demonstrate the effectiveness of the applied techniques in enhancing object detection performance in the specific context of the provided dataset.

#### 3.2.3. Dataset of Five Classes

The final dataset consists of a total of 600 photos with augmentation, which were partitioned into three subsets for training, validation, and testing. The training set contains 75% of the photos, amounting to 450 images, while the validation and testing sets each contain 15% of the photos, with 90 images in each set. 

Table 5 compares the performance of different YOLO models based on various Pascal metrics. Among the models, YOLOv7-X exhibits the highest overall performance with the highest A2, A5, and mAP values, showcasing its superior object classification and localization capabilities. YOLOv7-TINY also demonstrates strong performance across metrics, while some other models like YOLOv3 and YOLOv7-D6 show comparatively lower results in certain areas.

YOLOv7-X and YOLOv7-TINY exhibit superior performance compared to YOLOv7, YOLOv7-D6, and YOLOv7-W6 in terms of AP for most classes in Table 6. YOLOv7-X achieves high AP values across all classes, particularly excelling in A1 and A5. YOLOv7-TINY also demonstrates impressive AP scores, especially in A3 and A4. On the other hand, YOLOv7, YOLOv7-D6, and YOLOv7-W6 show comparatively lower AP values across the board. These findings indicate that YOLOv7-X and YOLOv7-TINY offer better accuracy and effectiveness in object detection and localization compared to the other YOLO variants. YOLOv7p-TINY, being a lightweight variant with reduced network parameters tailored for resource-constrained environments like robotics, can be a well-suited option for performing object detection tasks on robots.

**Figure 13:**
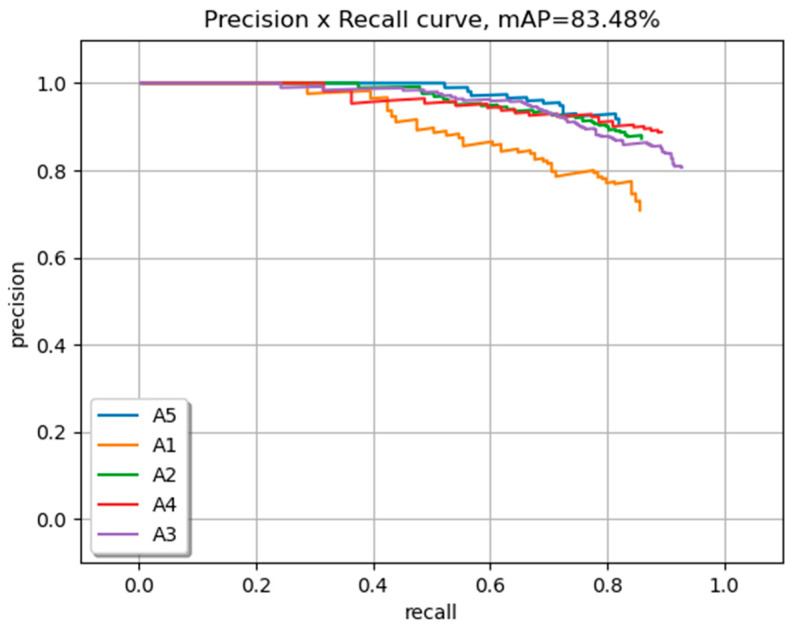
The mAP of five classes of tiny YOLOv7.

The mAP of YOLOv7-TINY is shown in Figure 13 from the detection of the training model.

**Figure 14:**
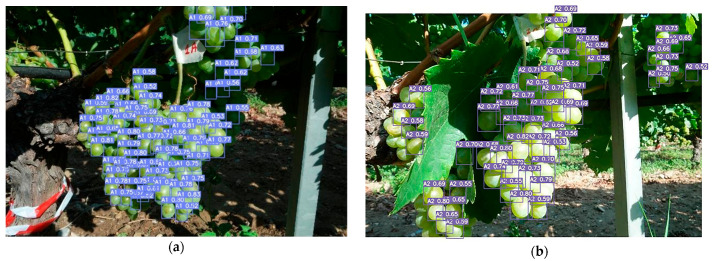

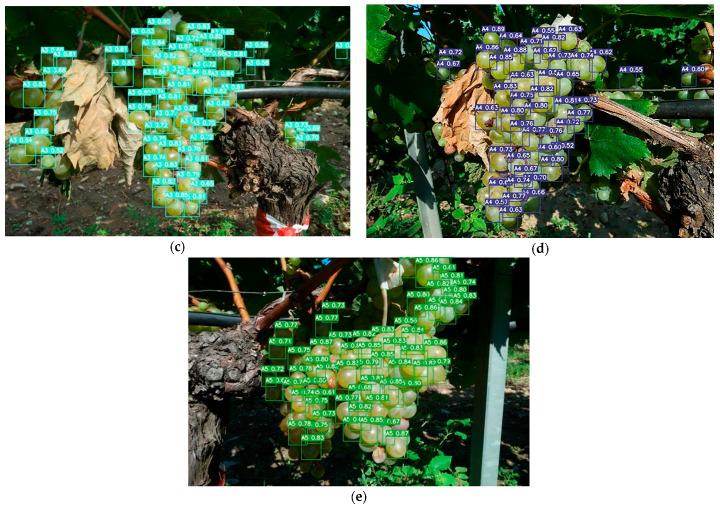
Detections with confidence 0.5 for classes (**a**) A1, (**b**) A2, (**c**) A3, (**d**) A4, and (**e**) A5.

In Figure 14, the same grape cluster is observed at different ripening stages, and its detection is performed using YOLOv7. The algorithm successfully detects and tracks the grape cluster across the various stages, showcasing its capability to consistently identify and localize the cluster throughout the maturation process.

## 4. Discussion

The algorithm’s effectiveness underwent rigorous testing and validation using an extensive dataset collected over a six-week period during grape ripening in the vineyards of Drama, Greece. This comprehensive dataset encompassed images captured from diverse angles and varying lighting conditions, ensuring that the algorithm’s performance remains robust and accurate even in real-world scenarios.

The successful detection of five distinct grape maturity stages supports the algorithm’s practical viability and potential for implementation in real-world applications. This achievement underlines its capacity to contribute significantly to grapevine management and cultivation practices.

Throughout the evaluation process, the algorithm’s performance was assessed using three distinct subsets, each shedding light on specific aspects of its capabilities. The first subset, focused on two classes, demonstrated the algorithm’s incremental improvement in terms of localization accuracy and overall object detection metrics. The subsequent introduction of a third class further bolstered the algorithm’s adaptability to accommodate varying maturity levels, enhancing its overall versatility.

In the case of the second subset, which comprised three classes, a combination of augmented data and advanced training techniques was employed to achieve notably improved results. The algorithm’s performance exhibited a substantial uptick in both detection and localization across different classes. In this context, an AP of 0.444 and a mAP of 0.792 demonstrate improved accuracy in classifying objects, specifically grape ripeness stages. These values indicate how well the algorithm identifies and localizes different grape maturity levels, offering a concise measure of its enhanced performance across classes. 

The final dataset, encompassing five classes, was meticulously divided into training, validation, and testing subsets. Through rigorous evaluation, two standout models, YOLOv7-X and YOLOv7-TINY, emerged as the frontrunners in terms of performance. These models consistently demonstrated higher AP 0.542 and 0.55 values, respectively across most classes, reaffirming their superiority in grape ripeness estimation in detected and localized berries.

## 5. Conclusions

The primary emphasis of this paper centers on estimating the maturity level of a white grape variety using high-resolution images. The objective is to facilitate informed grapevine management through precision agriculture methods, including determining the optimal harvest time and assessing grape maturity levels for future agricultural planning and operations. Based on the evaluation of the dataset, it can be concluded that the maturity estimation is at a satisfactory level. To further improve the implementation, it is recommended to include additional data from the next year’s harvest and retrain the model with this updated dataset. This will enable the model to better generalize maturity detection specifically for Assyrtiko grapes. As for future work, it is considered possible to expand the scope by incorporating other grape varieties into the maturity detection system. Future work will also consider different illumination conditions and enhance its findings by incorporating an updated version of YOLOv8 and integrating an architectural framework to achieve greater accuracy.

Furthermore, to enhance the capabilities of the autonomous harvesting system, deep learning techniques can be incorporated to estimate the values of important parameters such as pH, Brix (sugar content), and weight. By leveraging deep learning algorithms, the robot can make informed decisions regarding the optimal timing for harvesting. This integration of additional sensory data and advanced algorithms would enable the autonomous system to take accurate and precise actions during the harvesting process.

## Figures and Tables

**Table 1:** COCO metrics for subsets of 2 classes.

	AP	AP50	AP75	APmedium	APlarge	AR1	AR10	AR100
A	0.078	0.247	0.026	0.028	0.087	0.006	0.063	0.116
B	0.215	0.562	0.112	0.138	0.357	0.007	0.079	0.328

**Table 2:** Pascal metrics for subsets of 2 classes.

	A1	A5	mAP
A	0.101	0.391	0.246
B	0.568	0.572	0.571

**Table 3:** COCO metrics for subsets of 3 classes.

	AP	AP50	AP75	APmedium	APlarge	AR1	AR10	AR100
A	0.286	0.53	0.279	0.185	0.388	0.012	0.106	0.485
B	0.444	0.785	0.464	0.347	0.543	0.013	0.125	0.523

**Table 4:** Pascal metrics for subsets of 3 classes.

	A1	A3	A5	mAP
A	0.683	0.568	0.354	0.535
B	0.786	0.852	0.74	0.792

**Table 5:** COCO metrics.

	AP	AP50	AP75	APmedium	Aplarge	AR1	AR10	AR100
YOLOv7	0.401	0.728	0.487	0.322	0.529	0.011	0.11	0.491
YOLOv7-X	0.452	0.767	0.294	0.337	0.542	0.013	0.127	0.542
YOLOv7-TINY	0.48	0.829	0.525	0.369	0.562	0.013	0.128	0.55
YOLOv7-D6	0.337	0.601	0.343	0.241	0.409	0.012	0.111	0.457
YOLOv7-W6	0.342	0.58	0.365	0.269	0.393	0.012	0.113	0.454
YOLOv3	0.288	0.493	0.299	0.348	0.339	0.011	0.096	0.395
YOLOV5	0.337	0.611	0.331	0.256	0.401	0.012	0.107	0.442
Detectron2	0.174	0.395	0.129	0.286	0	0.007	0.068	0.264

**Table 6:** Pascal Metrics and Inference time per image.

	A1	A2	A3	A4	A5	mAP	Inference (ms)
YOLOv7	0.685	0.632	0.741	0.821	0.753	0.733	1.0
YOLOv7-X	0.788	0.811	0.740	0.467	0.869	0.770	1.5
YOLOv7-TINY	0.785	0.833	0.889	0.860	0.808	0.835	1.5
YOLOv7-D6	0.662	0.690	0.737	0.335	0.622	0.609	2.0
YOLOV5	0.782	0.527	0.760	0.446	0.574	0.618	1.6
YOLOV3	0.547	0.104	0.772	0.440	0.621	0.497	1.7
YOLOv7-W6	0.605	0.521	0.747	0.398	0.673	0.589	2.0

## Data Availability

Data are available on request due to restrictions.
